# Retrospective cohort study of neonatal blood transfusion in China

**DOI:** 10.1186/s12887-023-04225-5

**Published:** 2023-12-09

**Authors:** Ting Ma, Yang Sun, Qiushi Wang, Fenghua Liu, Kai Hua, Liqin Wang, Aowei Song, Wenhua Wang, Xinxin Xie, Jiangcun Yang, Xiling Li

**Affiliations:** 1 Department of Transfusion Medicine, Shaanxi Provincial People’s Hospital, 256 Youyi West Road, Xi’an, 710068 China; 2 Department of Data Center, Shaanxi Provincial People’s Hospital, Xi’an, 710068 China; 3 Department of Transfusion Medicine, Shengjing Hospital of China Medical University, Shenyang, 110004 China; 4 Department of Transfusion Medicine, The Fisrt Affiliated Hospital of Harbin Medical University, Harbin, 150000 China; 5 College of Life Sciences, Northwest University, Xi’an, 710068 China; 6 Department of Transfusion Medicine, Children’s Hospital Of ShanXi, Xinmin North Street, 13, Xinghualing District, Taiyuan, 030013 China

**Keywords:** Neonates, Blood transfusion, Cohort study

## Abstract

**Background:**

Blood transfusion therapy is extremely important for certain neonatal diseases, but the threshold for neonatal blood transfusion is not the same in different countries. Until now, clinical studies to determine the suitable threshold for newborns in China are lacking. Therefore, it is of high importance to establish a multi-center cohort study to explore appropriate transfusion thresholds for newborns in China.

**Methods:**

This retrospective cohort study investigated neonatal blood transfusion therapy administered from January 1, 2017 to June 30, 2018, with the aim of evaluating the effect of restricted and nonrestricted blood transfusion on neonatal health. The subjects were enrolled in 46 hospitals in China. A total of 5669 neonatal cases were included in the study. Clinical diagnosis and transfusion treatment of these neonates were collected and the data were retrospectively analyzed. The neonates were followed up 1 week and 1 month after leaving the hospital. The newborns’ and their mothers’ data were collected containing 280 variables in the database. The primary outcome of the study was mortality, and the secondary outcomes were complications, hospital stays, NICU hospital stays and hospital costs.

**Results:**

Results from the < 1500 g group showed that there was a higher mortality rate in the restricted transfusion group (11.41%) when compared with the non-restricted transfusion group (5.12%) (*P* = 0.000). Among the secondary outcomes, the restricted transfusion group had fewer costs. Results from the 1500-2500 g group showed that the mortality rates of the restricted and non-restricted transfusion groups were 3.53% and 4.71%, respectively, however there was no statistical significance between the two groups (*P* = 0.345). Among the secondary outcomes, the restricted transfusion group had fewer hospital stays, NICU hospital stays and hospital costs. The incidence of necrotizing enterocolitis was lower in the restricted transfusion group (OR, 2.626; 95% confidence interval [CI], 1.445 to 4.773; *P* = 0.003). The results from the ≥ 2500 g restricted transfusion group suggested that the mortality rate of (3.02%) was significantly lower than that of non-restricted transfusion group (9.55%) (*P* = 0.000). Among the secondary outcomes, the restricted transfusion group had fewer hospital stays and hospital costs. The incidence of retinopathy of prematurity was lower in the restricted transfusion group (OR, 4.624; 95% confidence interval [CI], 2.32 to 9.216; *P* = 0.000).

**Conclusions:**

Current transfusion protocols for newborns weighing less than 1500 g may be inappropriate and lead to higher mortality. The current transfusion threshold performed better for the other two weight groups.

**Supplementary Information:**

The online version contains supplementary material available at 10.1186/s12887-023-04225-5.

## Background

The development of neonatal hematopoietic system is not mature, and the condition changes rapidly during hospitalization. Transfusion therapy is widely used in neonatal patients. Due to these characteristics in very low birth weight (VLBW) neonates, transfusion rates during hospitalization can be as high as 80% [1–5]. Gestational age is known to be inversely proportional to the number of blood transfusions [6]. However, improper blood transfusion treatment also brings transfusion reactions. For example, excessive blood transfusion can lead to iron overload, heavy circulatory load, intraventricular hemorrhage and adverse reactions [7–11]. On the other hand, insufficient blood transfusion treatment will affect neonatal development and therapeutic effect disease treatment, and can lead to apnea, neurodysplasia or poor weight gain [12]. Therefore, it is extremely important to determine the threshold of neonatal blood transfusion treatment. A small group of countries including Britain, the United States and Australia have formulated clear neonatal blood transfusion programs [6, 13–15]. However, thresholds for neonatal transfusions vary from country to country. This may be due to regional, racial and ethnic differences in approach to medical conditions and treatment [16]. The standardization of neonatal blood transfusion protocols, however brings potential risks, especially for VLBW infants. A large number of studies have reported that strict blood transfusion thresholds increase the frequency of adverse neurologic events, reduce long-term brain volume, increase the incidence of periventricular leukomalacia and a series of intraventricular hemorrhage complications [17]. On the other hand, recent studies take the opposite view, particularly in two large cohort studies that found no difference in mortality or complications between lower and higher blood transfusion thresholds [18, 19]. Doctors in some parts of China refer to clinical experience and foreign neonatal transfusions standards when conducting neonatal transfusion therapy. The recommended threshold for neonatal blood transfusion treatment in China is otherwise taken from Practical Neonatology (5th edition) [20]. For neonates weighing less than 1500 g, the recommended transfusion threshold is as published in the UK in 2015. The other neonatal-weight groups’ transfusions were judged comprehensively according to clinical experience, normal range of Hb range. Up until now, clinical studies to determine a suitable threshold for Chinese newborns are lacking. Therefore, it is important to establish a multi-center retrospective cohort study to evaluate whether the blood transfusion scheme is suitable for newborns in China.

In this study, a multi-center retrospective cohort study was established to observe the transfusion treatment status, clinical symptoms, signs and other variables in neonates during hospitalization, and at 1 week and 1 month follow-up points after discharge. The cohort involved 5669 transfused newborns from 46 hospitals in 21 provinces and 7 regions of China. Each case contained 280 variables, out of a total of 2.98 million variables. The main objectives of this cohort study included the following: Newborns with different body weights were defined as restricted blood transfusion (RBT) and non-restricted blood transfusion (NRBT) cohorts respectively according to the recommendations on neonatal transfusion thresholds in Practical Neonatology (5th edition) (Supplementary Table 1), so that the applicability of this protocol in Chinese neonates could be evaluated according to outcome. From this, appropriate neonatal transfusion thresholds and neonatal transfusion protocols were sought for future guidance in Chinese hospitals. This study was approved by the Ethics Committee of the Institutional Evaluation Committee of Shaanxi Provincial People's Hospital (NO: 2020-R001).

## Methods

### Cohort participants

**Fig. 1:**
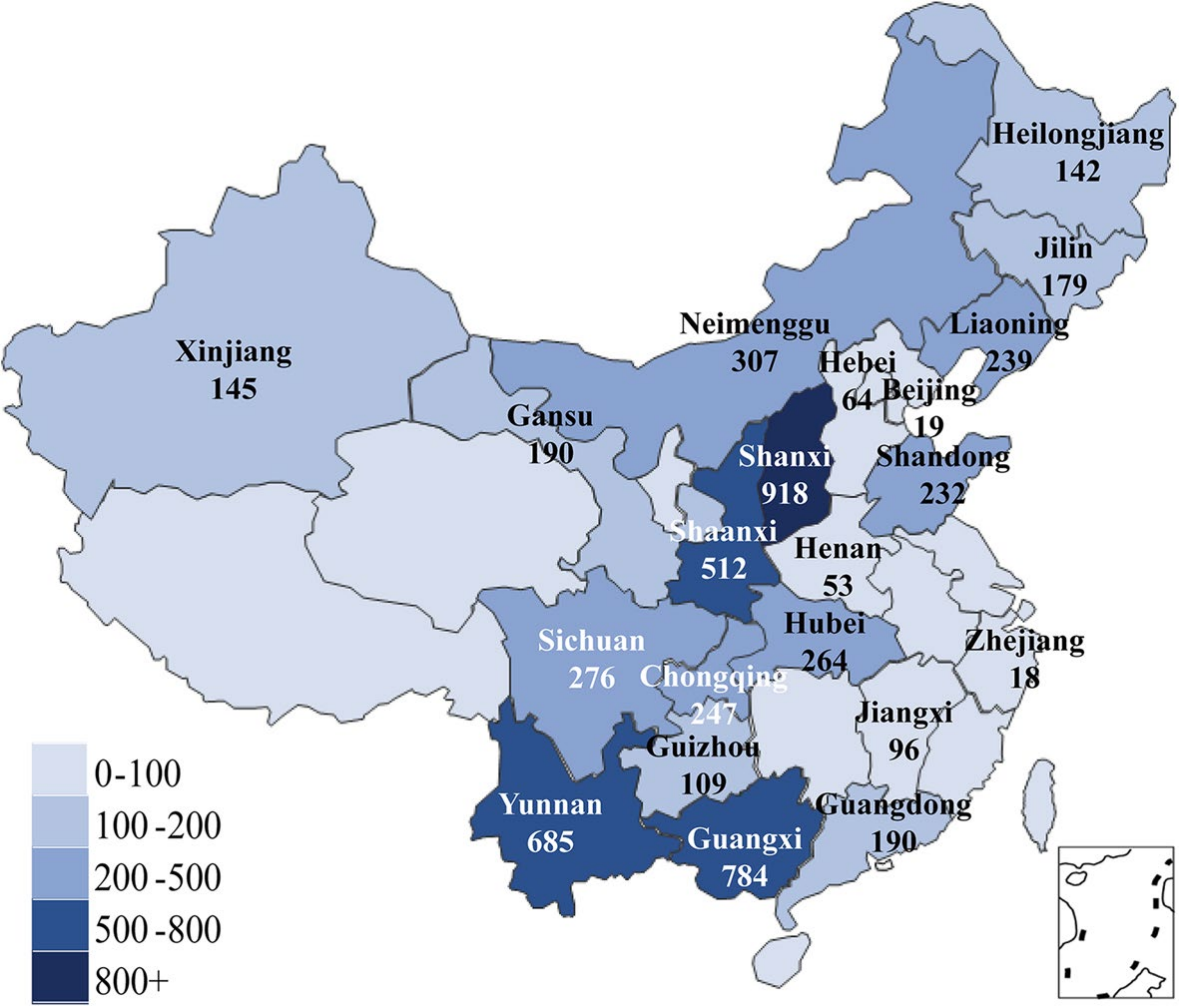
The geographical distribution of neonatal blood transfusion cohort in China

Diagnosis and treatment data from blood transfusion inpatients was taken from 46 hospitals in 21 provinces in China. A multi-center retrospective study method was carried out to systematically investigate blood transfusion efficacy in newborns. A unified spreadsheet was used to collect data from forty-six hospitals covering 21 provinces and cities (Beijing, Gansu, Guangdong, Guangxi, Guizhou, Hebei, Henan, Heilongjiang, Hubei, Jilin, Jiangxi, Liaoning, Inner Mongolia, Shandong, Shanxi, Shaanxi, Sichuan, Xinjiang, Yunnan, Zhejiang, Chongqing, shown in Fig. 1. Microsoft® Excel® 2019MSO). The data spreadsheets were compiled in Shaanxi Provincial People's Hospital, for unified collation and analysis. The database included 5669 hospitalized newborns with blood transfusion from January 2017 to June 2018. There were 280 variables in each case, including newborn mother information and newborn blood transfusion treatment information (as shown in Supplementary Table 2).

**Fig. 2:**
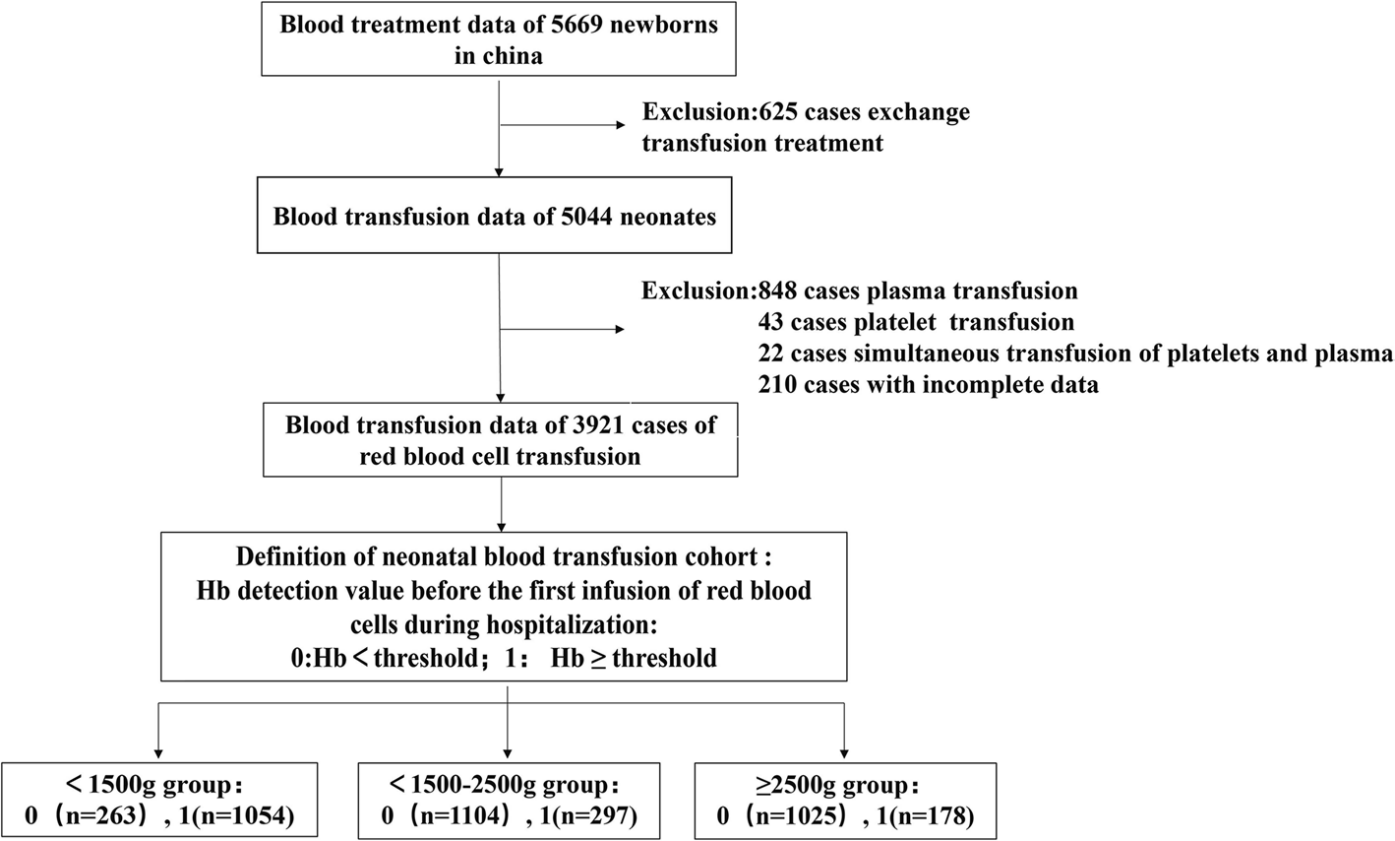
Flowchart demonstrates the steps of cohort

Among the 5669 newborns, 625 underwent transfusion exchange treatment, 848 underwent simple plasma transfusion, 43 received platelet transfusion only, while 22 received transfusion of both plasma and platelets. 210 newborns with incomplete data were excluded. Altogether, for this study 1.8 million items of data from 3921 newborns, who underwent red blood cell transfusion were analyzed (shown in Fig. 2).

In this cohort study, 3921 newborns infused with red blood cells were divided into three groups according to their birth weight, namely < 1500 g, 1500-2500 g, and ≥ 2500 g. Transfusion intervention was performed according to the threshold of neonatal blood transfusion in the fifth edition of Practical Neonatology (Supplementary Table 1), and the newborns’ Hb value before first blood transfusion was recorded. An Hb of lower than the threshold was defined as the restricted blood transfusion group (RBT = 0), while an Hb higher than the threshold was defined as the non-restricted blood transfusion group (NRBT = 1). Three neonatal blood transfusion groups, organized by different weights were then established.

### Blood sampling

Among the variables observed, 5 blood transfusion time points, transfusion methods and more blood index detection points were included, so as to evaluate blood transfusion’s effect over time effectively. Blood samples were collected and tested according to strict guidelines. A blood routine index test was taken using 10 results after admission. If the detection time point of the neonate was less than the set number of times, all available results were included.

### Cohort follow-up

The first statistical analysis of data included the variables during hospitalization and the follow-up results of 1 weeks and 1 months after discharge. The survival of all newborns was followed up from 1 week to 1 month after discharge.

### Statistical analysis

Descriptive statistics are presented as median with an inter-quartile range for continuous data according to the characteristics of the data distribution. Categorical variables were summarized as counts and percentages. A *p*-value of less than 0.05 was considered statistically significant. Independent t test, Mann–Whitney test or χ2 test were used to compare the characteristics of the groups. *P* < 0.05 was considered to be statistically signifificant. Statistical analysis was performed using IBM SPSS version 24.

Univariate logistic regression models estimated correlations between mortality, ROP, PVL, BPD, NEC, and grouping. A *p*-value of less than 0.05 was considered statistically significant. A multivariate logistic regression model was then used to estimate the association between mortality and a set of covariables. Factors that differed at baseline and previous clinical experience were included in the model, including grouping, birth weight, birth length, head circumference, number of ventilated newborns, admission haemoglobin, and number of red cell transfusions. *P*-values less than 0.05 were considered statistically significant. Odds ratios (OR) and 95% confidence intervals (CI) were calculated for each risk factor.

## Results

In the three cohorts with different weights, the baseline distribution of mothers' baseline age, delivery mode, multiple births, parity, pregnancy times and accompanying complications were not statistically different (Table 1). The baseline characteristics of newborns in the three groups were basically also similar in terms of sex, birth weight, birth length, birth head circumference, admission weight, Apgar score, and number of concomitant diseases. The < 1500 g group had differences in Apgar at 5 min, 10 min and ventilator use. There were differences in gender, birth length, admission weight and ventilator use in the 1500-2500 g group. The birth weight, Apgar score and ventilator use were significantly different in the ≥ 2500 g group (Table 2).

**Table 1:** Basic demographic characteristics of mothers in the neonatal blood transfusion cohort

		** < 1500 g**			**1500-2500 g**			** ≥ 2500 g**		
		**RBT**	**NRBT**	***P***	**RBT**	**NRBT**	***P***	**RBT**	**NRBT**	***P***
**Total (n)**		263	1054		1104	297		1025	178	
**Age**		30 (19–44)	30 (18–42)	0.194	30 (16–45)	30 (18–47)	0.659	30 (17–44)	30 (14–44)	0.658
**Mode of delivery(n)**	Natural delivery	110	471	0.755	345	111	0.076	443	77	0.486
	Midwifery	4	8		12	5		21	4	
	Cesarean section	148	574		747	181		560	97	
	Unknown	1	1		0	0		1	0	
**Number of births(n)**	Single fetus	166	734	0.062	771	215	0.324	962	164	0.773
	Multiple births	92	309		321	78		48	7	
	Unknown	5	11		12	4		15	7	
**Pregnancy times(n)**	1	68	387	0.012	375	107	0.312	345	64	0.640
	2	85	302		304	90		331	55	
	3 times or more	94	318		374	92		332	54	
	Unknown	16	47		51	8		17	5	
**Parity(n)**	1	112	484	0.282	447	143	0.065	474	89	0.600
	2	121	450		522	119		462	66	
	3 times or more	21	77		96	28		71	18	
	Unknown	9	43		39	7		18	5	
**Number of mothers with complications (n)**		117	453	0.009	401	118	0.944	276	39	0.149

^*^RBT Restricted blood transfusion, *NRBT* Non-restricted blood transfusion

**Table 2:** Basic demographic characteristics of newborns in the neonatal blood transfusion cohort [M(max, min)]

		** < 1500 g**			**1500-2500 g**			** ≥ 2500 g**		
		**RBT**	**NRBT**	***P***	**RBT**	**NRBT**	***P***	**RBT**	**NRBT**	***P***
**Total (n)**		263	1054		1104	297		1025	178	
**Gender**	Male	148	552	0.257	626	189	0.032	610	107	0.892
	Female	115	502		478	108		415	71	
**Birth weight (g)**		1235 (530–1490)	1226 (630–1498)	0.817	1813 (1500–2480)	1760 (1500–2490)	0.147	3025 (2500–5500)	3175 (2500–8600)	0.002
**Birth length (cm)**		38 (27–54)	38 (29–53)	0.486	43 (28–60)	43 (32–53)	0.017	50 (32–57)	50 (42–56)	0.044
**Head circumference (cm)**		28 (21–47)	28 (18–40)	0.992	30 (21–41)	30 (21–49)	0.766	34 (23–48)	34 (31–44)	0.043
**Admission weight (g)**		1265	1250	0.684	1870	1750	0.001	3040	3100	0.291
**Apgar score**	1 min	8	8	0.179	9	8	0.084	9	9	0.007
	5 min	9	9	0.005	9	9	0.050	10	9	0.012
	10 min	10	9	0.023	10	10	0.192	10	10	0.010
**No. of diseases associated**		4 (0–10)	4 (0–11)	0.414	4 (0–14)	4 (0–12)	0.149	2 (0–8)	2 (0–6)	0.531
**No. of ventilated newborns (%)**		219 (83.27)	720 (68.31)	0.000	571 (51.72)	177 (59.60)	0.033	285 (27.83)	74 (41.34)	0.000

^*^RBT Restricted blood transfusion, *NRBT* Non-restricted blood transfusion

The investigation of blood transfusion treatment in the < 1500 g group showed that compared with the NRBT group, the RBT group had lower levels of Hb at the time of admission and hospitalization. The number of infusions and average total infusion volume were larger in the RBT group, but the average infusion volume per infusion was the same between the two groups (Table 3). Among secondary outcome indicators, the RBT group had fewer costs. The main outcome indicators showed that there was a statistical difference between the two groups. The implementation of this standard significantly increased the risk of death. The mortality rate in the RBT group (11.41%) was higher than that in the NRBT group (5.12%) (Tables 4, 5).

**Table 3:** Treatment of blood transfusion by restricted blood transfusion and non-restricted blood transfusion

		** < 1500 g**			**1500-2500 g**			** ≥ 2500 g**		
		**RBT**	**NRBT**	***P***	**RBT**	**NRBT**	***P***	**RBT**	**NRBT**	***P***
**Hb level** **(g/L)**	**Admissions (M)**	109	157	0.000	133	165	0.000	110	159	0.000
	**Minimum during hospitalization (M)**	82	95	0.000	94	111	0.000	94	121	0.000
	**Discharge(M)**	111	117	0.150	116	126	0.000	121	134	0.000
**Red blood cell infusion**	**No. of infusions (M)**	2	2	0.000	1	1	0.351	1	1	0.798
	**Average total infusion volume (mL)**	60	50	0.000	46	44	0.147	60	60	0.131
	**Average infusion volume per infusion (mL)**	26	27	0.843	35	31	0.000	50	50	0.015
	**Mean infusion per kilogram of body weight (mL)**	10	10	0.867	9	9	0.064	8	8	0.239
**Plasma infusion**	**No. of infusion cases (%)**	53 (20.15)	289 (27.42)		126 (11.41)	143 (48.15)		140 (13.66)	56 (31.46)	
	**No. of infusions (M)**	1	2	0.113	1	2	0.039	1	2	0.200
	**Average total infusion volume (mL)**	30	34	0.998	47	57	0.146	71	93	0.023
	**Average infusion volume per infusion (mL)**	20	20	0.053	30	28	0.142	50	50	0.051
	**Mean infusion per kilogram of body weight (mL)**	9	8	0.022	8	8	0.803	7	8	0.240
**Plt infusion**	**No.of infusion cases (%)**	13 (4.94)	35 (3.32)		24 (2.17)	12 (4.04)		30 (2.93)	9 (5.06)	
	**No. of infusions (M)**	1	1	0.161	1	1	0.577	1	1	0.805
	**Average total infusion volume (mL)**	28	23	0.388	40	38	0.987	60	80	0.429
	**Average infusion volume per infusion (mL)**	20	20	0.809	35	31	0.688	58	50	0.582
	**Mean infusion per kilogram of body weight (mL)**	9	9	0.971	10	9	0.945	9	8	0.756

^*^RBT Restricted blood transfusion, *NRBT* Non-restricted blood transfusion

**Table 4:** Analysis of main outcome indicators of neonatal blood transfusion

		** < 1500 g**						**1500-2500 g**						** ≥ 2500 g**					
		**RBT**	**NRBT**	**OR**	**CI**		***P***	**RBT**	**NRBT**	**OR**	**CI**		***P***	**RBT**	**NRBT**	**OR**	**CI**		***P***
**Total number (n)**		263	1054					1104	297					1025	178				
**Death**	**Total n (%)**	30 (11.41)	54 (5.12)	0.416	0.260	0.666	0.000	39 (3.53)	14 (4.71)	1.374	0.735	2.569	0.345	31 (3.02)	17 (9.55)	3.532	1.905	6.547	0.000
	**During hospitalization n (%)**	11 (4.18)	25 (2.37)	0.557	0.27	1.146	0.112	8 (0.72)	5 (1.68)	2.346	0.762	7.224	0.137	9 (0.88)	6 (3.37)	3.938	1.384	11.203	0.010
	**Follow-up for 1 week n (%)**	15 (5.7)	22 (8.37)	0.595	0.381	0.930	0.023	20 (1.81)	7 (2.36)	1.308	0.548	3.124	0.545	17 (1.66)	8 (4.49)	2.79	1.186	6.567	0.019
	**Follow-up for 1 month n (%)**	4 (1.52)	7 (2.66)	0.433	0.126	1.490	0.184	11 (1.00)	2 (0.67)	0.674	0.149	3.056	0.609	5 (0.49)	3 (1.69)	3.497	0.828	14.765	0.088

^*^RBT Restricted blood transfusion, *NRBT* Non-restricted blood transfusion

**Table 5:** Analysis of secondary outcome indicators of neonatal blood transfusion

	** < 1500 g**			**1500-2500 g**			** ≥ 2500 g**		
	**RBT**	**NRBT**	***P***	**RBT**	**NRBT**	***P***	**RBT**	**NRBT**	***P***
**Hospital stays (d,M)**	38	40	0.074	22	26	0.002	10	14	0.000
**NICU hospital stays (d,M)**	30	29	0.547	9	16	0.000	0	0	0.186
**Hospitalization costs (Yuan, M)**	53,954	68,573	0.000	36,727	51,365	0.000	17,891	22,959	0.022

^*^RBT Restricted blood transfusion, *NRBT* Non-restricted blood transfusion

The study of blood transfusion treatment in the 1500-2500 g group suggested that compared with the NRBT group, the RBT group had lower levels of Hb at the time of admission, hospitalization and discharge. There were no differences in other blood transfusion treatments between the two groups. Among the secondary outcome indicators, the RBT group had less hospitalization time, NICU time, and hospital costs. The incidence of necrotizing enterocolitis (NEC) was lower in RBT group (Table 6). The mortality rate of RBT group (3.53) was lower than that of NRBT group (4.71) (Tables 4, 5), but there was still no statistical significance between the two groups.

**Table 6:** Analysis of transfusion related complications in neonates

	**RBT**	**NRBT**	***P***	**OR**	**CI**	
					**Lower**	**Upper**
**ROP n(%)**						
** < 1500 g**	28(10.65)	128(12.14)	0.593	1.16	0.752	1.789
**1500-2500 g**	39(3.53)	13(4.38)	0.491	1.25	0.658	2.373
** ≥ 2500 g**	20(1.95)	15(8.43)	0.000	4.624	2.32	9.216
**PVL n(%)**						
** < 1500 g**	5(1.90)	21(1.99)	1.000	1.049	0.392	2.808
**1500-2500 g**	28(2.54)	5(1.68)	0.519	0.658	0.252	1.719
** ≥ 2500 g**	9(0.88)	1(0.56)	1.000	0.638	0.08	5.065
**BPD n(%)**						
** < 1500 g**	48(18.25)	167(15.84)	0.352	0.843	0.592	1.201
**1500-2500 g**	56(5.07)	10(3.37)	0.280	0.652	0.329	1.294
** ≥ 2500 g**	8(0.78)	4(2.25)	0.088	2.922	0.871	9.81
**NEC n(%)**						
** < 1500 g**	11(4.18)	55(5.22)	0.635	1.261	0.651	2.445
**1500-2500 g**	28(2.54)	19(6.40)	0.003	2.626	1.445	4.773
** ≥ 2500 g**	9(0.88)	0(0.00)	0.371			
**IVH n(%)**						
** < 1500 g**	88(33.46)	339(32.16)	0.713	0.943	0.708	1.256
**1500-2500 g**	247(22.37)	54(18.18)	0.131	0.771	0.556	1.069
** ≥ 2500 g**	110(10.73)	16(8.99)	0.596	0.822	0.474	1.425

^*^RBT Restricted blood transfusion, *NRBT* Non-restricted blood transfusion

Blood transfusion treatment in the ≥ 2500 g group suggest that compared with the NRBT group, the RBT group had lower levels of Hb at the time of admission, hospitalization and discharge. There was no significant difference between the two groups in the methods of red blood cells, plasma and platelet transfusion. Among the secondary outcome indicators, the restricted transfusion group had less hospitalization time and hospital costs. The incidence of retinopathy of prematurity (ROP) was lower in the RBT group (Table 6). The death outcome of the two groups showed that the mortality rate of RBT group (3.02) was significantly lower than that of the NRBT group (9.55) (Tables 5, 6).

Multivariate logistic regression model analysis showed that mortality rate of NRBT group was 0.52 times than that of RBT group (95% CI: 0.282–0.959, *P* < 0.05) in the < 1500 g group. There was no significant difference between the two groups in the 1500–2500 g group. The mortality rate of the NRBT group was 3.052 times higher than that of RBT group (95% CI: 1.128–8.255, *P* < 0.05) in the ≥ 2500 g group, and the application of ventilator intervention had a protective factor against death. The results showed that neonatal mortality with different weights were consistent with those of the univariate analysis (Supplementary Table 3).

## Discussion

This multi-center cohort study was the largest epidemiological analysis of neonatal blood transfusion practice in China to evaluate the application of blood transfusion thresholds to newborns. Included were basic information regarding transfused newborns and mothers and detailed information on the blood transfusion treatment given, examination results, complications and mortality. In the < 1500 g group, the RBT mortality rate was significantly higher than the NRBT group, and the risk of death was increased. We disagree whether this threshold was applicable to the newborns in < 1500 g group. There was no difference in mortality between the 1500-2500 g group, but the hospital stays and costs were significantly lower when the lower restrictive threshold was implemented. The incidence of acute NEC was lower. The outcome of the RBT group showed better performance. The mortality rate of RBT in > 2500 g group was significantly lower than that in the NRBT group. The threshold treatment scheme was protective to newborns, and the incidence of ROP was lower and produced fewer hospital stays and fewer medical costs. This group also showed better discharge outcome in the RBT threshold group.

It has been reported that different blood transfusion thresholds should be adopted for newborns with different weights, and a higher threshold should be carried out in NRBT for newborns with very low weights, so as to ensure the development of the nervous system. More and more studies have shown that the adverse effects of RBT on newborns have inconsistent results, especially for low birth weight infants and newborns with severe diseases [21–25]. Some recently published prospective studies show that there is no difference in mortality between restricted and non-restricted transfusion thresholds for premature infants [18, 19]. Some researchers believe that a broader blood transfusion threshold is not beneficial, citing increased mortality and complications [26–29]. However, it is obvious that the threshold of neonatal blood transfusion is not consistent among countries, which may be one of the reasons for different research results. The results of this study showed that the overall mortality rate was significantly increased in the < 1500 g body weight group using the current blood transfusion threshold. The other two groups showed a positive effect in reducing mortality, especially in the > 2500 g group, while RBT showed the same trend.

Transfusion will disturb the microcirculation regulation of the body and may lead to a series of transfusion-related complications [30]. Blood transfusion has been correlated with death, bronchopulmonary dysplasia (BPD), periventricular leukomalacia (PVL), necrotizing enterocolitis (NEC), intraventricular hemorrhage (IVH) and retinopathy of prematurity (ROP). The long-term results of neurological development under different blood transfusion criteria have shown contradictory results in a large number of studies [17–19]. The results of the previous studies show that the incidence of early ROP in RBT group is slightly lower. Frequent and large-scale blood transfusion may be related to increased risk of inflammatory state [24, 31]. Blood transfusion rates are higher in premature infants than in full-term infants, accordingly results here also found that the incidence of ROP in very low birth weight (< 1500 g) newborns was higher than 10%. Moreover, the incidence of ROP with restricted blood transfusion regimens was lower in the group with body weights of ≥ 2500 g.

Results on the incidence of NEC showed that the incidence was lower when a restrictive blood transfusion threshold was applied [31]. However, Patel et al. concluded that Anemia will increase the risk of NEC [32]. A prospective cohort study conducted by Ozcan B. in 2020 showed that the level of L-fatty acid binding protein in premature infants with anemia increased, suggesting that anemia aggravated intestinal injury [33]. Meta-analysis of several studies showed contradictory results about blood transfusion and NEC incidence [34]. The incidence of NEC was lower in our study on newborns with a lower blood transfusion threshold of 1500 g-2500 g weight. There was no difference in the incidence of NEC between the other weight groups whether restricted or unrestricted transfusions were performed.

Research into IVH caused by blood transfusion also show different conclusions. Most research results show that blood transfusion will increase the occurrence of IVH, however a few reports suggest that preventing anemia is beneficial to IVH. This study’s results were consistent with the ETTNO and TOPs studies outlined above, and no significant difference was found in different blood transfusion thresholds for IVH. Higher blood transfusion thresholds did not reduce the incidence of IVH [7, 17].

In literature concerning red blood cell transfusion dosage, neonatal red blood cell transfusion dosage is usually reported at between 5—20 mL/kg worldwide. BCSH guidelines stipulate that the infusion amount of neonatal red blood cells should be 10-20 mL per kilogram. When accompanied by bleeding and severe anemia, the infusion amount should be appropriately increased, however there is no recommended amount for appropriate increase. At present, the recommended red blood cell infusion dose for newborns in China is generally small at (10—20) ml/kg per time, while for premature infants, especially at very low birth weights, it is generally (5—15) ml/kg per time. The results of this study showed that the infusion volume for different body weight groups was (8–10) ml/kg per time. There was no difference in the comparison between restricted and unrestricted transfusions and different weight groups, so different blood transfusion thresholds had no effect on the infusion volume per kilogram of body weight, and the blood transfusion volume per kilogram of body weight was consistent between the two groups. Chinese doctors show a high degree of consistency in the application of blood transfusion volume. The number and total amount of blood transfusions in the < 1500 g group were significantly higher than those in the unrestricted group because of the lower Hb value in the restricted group.

## Conclusions

In summary, we question whether a recommended blood transfusion threshold is suitable for Chinese newborns. The results show that the 1500-2500 g newborns had no significant difference in mortality and no complications was found between the two groups except for the incidence of NEC. The lower blood transfusion threshold played a protective role in NEC. The shortened newborns’ hospital stays and reduced treatment cost were also encouraging. Restricted blood transfusion thresholds in newborns (≥ 2500 g) significantly reduced mortality, and the incidence of ROP was lower than that in non-restricted blood transfusion groups. The hospital stays and treatment costs were also reduced. It is worth noting that although there was no difference in complications relating to blood transfusions in very low birth weight infants, lower blood transfusion thresholds lead to a significant increase in mortality. From these results, it is clear that we need to find a more suitable blood transfusion scheme for very low birth weight infants (< 1500 g).

### Strengths and limitations

This cohort study had some limitations. Firstly, the cohort is based on retrospective data, so there were differences in the amount of cases and baseline data between the restricted blood transfusion group and unrestricted blood transfusion group. Secondly, the observation outcome index in this paper includes a limited follow-up study of 1 week and 1 month after discharge. A more satisfactory endpoint would be a comprehensive long-term evaluation table from prospective newborn to 3-year-old exit. Thirdly, due to the differences in geographical location and economic situation, a limited amount of data was unavailable, though by way of the large sample involved here, this was of limited consequence. This report concludes that an adjustment to the inappropriate blood transfusion scheme and a blood transfusion threshold scheme that better meets requirements would be beneficial to the health of Chinese newborns undergoing blood transfusions.

### Supplementary Information

**Additional file 1: Supplementary Table 1.** Neonatal blood transfusion threshold (The fifth edition of Practical Neonatology) Hb (g/L).

**Additional file 2: Supplementary Table 2.** Data collection of neonatal blood transfusion cohort: variables of neonatal mother and neonatal groups.

**Additional file 3: Supplementary Table 3.** Risk analysis of mortality using multiple regression model.

## Data Availability

We warmly welcome the cooperation for the cohort study of neonatal blood transfusion in China and make full use of the data. Since the research is still undergoing and the information used in this study relate to personal privacy, the database cannot be open in the public domain for free, but researchers interested in collaboration and further information are welcome to contact the corresponding authors via email [yjc65@sina.com].

## Abbreviations

VLBW: Very low birth weight
TA-GVHD: Transfusion-related graft host disease
BPD: Bronchopulmonary dysplasia
PVL: Periventricular leukomalacia
NEC: Necrotizing enterocolitis
IVH: Intraventricular hemorrhage
ROP: Retinopathy of prematurity
