# Supplementary material for: Retrospective cohort study of neonatal blood transfusion in China
Source: BMC Pediatr. 2023 Dec 9;23:621. doi: 10.1186/s12887-023-04225-5 (PMC10709978; doi:10.1186/s12887-023-04225-5)
Supplement: Supplementary file 1 — Additional file 1: Supplementary Table 1. Neonatal blood transfusion threshold (The fifth edition of Practical Neonatology) Hb (g/L). [file 12887_2023_4225_MOESM1_ESM.docx]

**Supplementary Table 1** Neonatal blood transfusion threshold (The fifth edition of Practical Neonatology) Hb (g/L)

| Postnatal age | < 1500g | | 1500-2500g | >2500g |
| --- | --- | --- | --- | --- |
|  | Respiratory Support | No Respiratory Support |  |  |
| 1-3 | 115 | 100 | 150 | 150 |
| 4-7 | 115 | 100 | 135 | 140 |
| 8-14 | 100 | 85 | 120 | 125 |
| ≥15 | 85 | 75 | 120 | 118 |
