# Supplementary material for: Retrospective cohort study of neonatal blood transfusion in China
Source: BMC Pediatr. 2023 Dec 9;23:621. doi: 10.1186/s12887-023-04225-5 (PMC10709978; doi:10.1186/s12887-023-04225-5)
Supplement: Supplementary file 2 — Additional file 2: Supplementary Table 2. Data collection of neonatal blood transfusion cohort: variables of neonatal mother and neonatal groups. [file 12887_2023_4225_MOESM2_ESM.docx]

**Supplementary Table 2 Data collection of neonatal blood transfusion cohort: variables of neonatal mother and neonatal groups**

| **Mother variable** | **Neonatal variable** | | | |
| --- | --- | --- | --- | --- |
|  | **Baseline** | **Blood test index** | **Treatment and observation indicators** | **Outcome indicator** |
| Provinces | Gender | Neonatal blood group | Operation (8 species) | Discharge weight |
| Name of hospital | Date of birth | Routine blood index (10 times test)(RBC/HB/HCT/WBC/PLT) | Resuscitation Methods in Delivery Room (9 species) | Hospitalization days (days) |
| Age | Date of admission | Liver function index (5 times test)(TBIL/DBIL/ALT/TP) | Ventilator application (Yes/No) | Hospitalization expenses |
| Mode of delivery (Natural delivery/ Midwifery/Cesarean section) | Date of discharge | Coagulation index (5times test)(APTT/PT/INR/FIB) | Postpartum umbilical cord clipping time(0-30s/1-60s/1-2min/2min/unknow) | Days in NICU (days) |
| Number of births (1/ ≥2 ) | Birth weight | Blood gas index (10 times test) (PH/PO2/PCO2/LAC) | Blood transfusion treatmen t( time/volume/type(15 blood products)) | Main diagnosis of discharge (14 species) |
| Pregnancy times (1/2/≥3) | Birth length |  | Adverse events of blood transfusion (8 species) | Outcome (cure and improvement/transfers/death) |
| Parity(1/2/≥3) | Birth head circumference |  | Adverse events of blood transfusion (8 species) | Complications (12 species) |
| Maternal complication (11 species) | Admission weight |  | Evaluation of curative effect after blood transfusion(4 species) | Causes of death (9 species) |
|  | Apgar score (1-5-10) |  | Evaluation of curative effect after blood transfusion(4 species) | Follow-up after discharge (1W/1M/3Y) |
|  | Neonatal Admission Accompanying Diseases (15 species) |  | Exchange transfusion therapy (time/volume/blood products /blood type） | Bailey scale |

Maternal complication (11 species) = 1 no 2 respiratory system disease 3 circulatory system disease 4 digestive system disease 5 blood disease 6 childbirth disease 7 reproductive system infection 8 Maternal and infant blood type incompatibility 9 uterine disease 10 placental comorbidities 11 other diseases. Neonatal Admission Accompanying Diseases (15 species) = 1 premature infant 2 low birth weight infant 3 sepsis 4 neonatal necrotizing colitis 5 neonatal bleeding 6 neonatal hemolysis 7 neonatal hyperbilirubinemia (non-immune factor) 8 neonatal anemia 9 thrombocytopenia 10 neonatal disseminated intravascular bleeding 11 neonatal pneumonia 12 birth asphyxia 13 neonatal bronchopulmonary dysplasia 14 neonatal respiratory distress syndrome 15 other neonatal diseases. Operation (8species) = 1 no 2 PICC 3 umbilical vein catheterization 4 blood exchange 5 lumbar puncture 6 blue light irradiation 7 cardiovascular surgery 8 other operations. Resuscitation Methods in Delivery Room (9 species)= 1 normobaric oxygen therapy 2 balloon pressurized oxygen therapy 3 endotracheal intubation balloon pressurized oxygen therapy 4 chest compressions 5 trachea cannula 6 epinephrine 7 volume expansion 8 naloxone 9 unknown. Type(15 blood products) =1 red blood cell (leukocyte-filtered, irradiated) 2 red blood cell (non-leukocyte-filtered, irradiated) 3 red blood cell (non-leukocyte-filtered, nonirradiated) 4 red blood cell ( irradiated) 5 red blood cells (leukocyte-filtered) 6 fresh frozen plasma 7 fresh frozen plasma (virus inactivated) 8 frozen plasma 9 apheresis platelets (leukocyte-filtered) 10 apheresis platelets (non-leukocyte-filtered) 11 cryoprecipitate 12 whole blood 13 CMV antibody testing of blood products (positive) 14 CMVantibody testing of blood products (negative) 15 CMV antibody testing of blood products (unknown). Adverse events of blood transfusion (8 species) = 1 hemolysis reaction 2 coagulation dysfunction 3 circulation overload 4 metabolic disorder 5 acute necrotizing enterocolitis 6 hypothermia 7 hypocalcemia 8 acute lung injury. Evaluation of curative effect after blood transfusion(4 species) = color of skin, mental state, feeding, heart rate reduction(1 obvious, 2 improved, 3 normal, 4 no change). Main diagnosis of discharge (14 species)=1 premature infant 2 low birth weight infant 3 sepsis 4 neonatal necrotizing colitis 5 neonatal bleeding 6 neonatal hemolysis 7 neonatal hyperbilirubinemia (non-immune factor) 8 thrombocytopenia 9 neonatal disseminated intravascular bleeding 10 neonatal pneumonia 11 neonatal asphyxia 12 neonatal bronchopulmonary dysplasia 13 neonatal respiratory distress syndrome 14 other neonatal diseases. Complications (12 species)= 1 none 2 retinopathy of prematurity 3 acute necrotizing enterocolitis 4 chronic bronchopulmonary dysplasia 5 intracranial hemorrhage 6 neonatal periventricular leukomalacia 7 cholestasis syndrome 8 hepatitis 9 neurodevelopmental disorder 10 neonatal encephalopathy 11 coagulation dysfunction 12 others. Causes of death (9 species) = 1 sepsis 2 pulmonary hemorrhage 3 pneumonia 4 respiratory distress syndrome 5 asphyxia 6 bronchopulmonary dysplasia 7 hypoxic ischemic encephalopathy 8 neonatal necrotizing enterocolitis 9 others.
