# Supplementary material for: Retrospective cohort study of neonatal blood transfusion in China
Source: BMC Pediatr. 2023 Dec 9;23:621. doi: 10.1186/s12887-023-04225-5 (PMC10709978; doi:10.1186/s12887-023-04225-5)
Supplement: Supplementary file 3 — Additional file 3: Supplementary Table 3. Risk analysis of mortality using multiple regression model. [file 12887_2023_4225_MOESM3_ESM.docx]

**Supplementary Table 3** **Risk analysis of mortality using multiple regression model**

|  |  | *P* | OR | CI | |
| --- | --- | --- | --- | --- | --- |
|  |  |  |  | Lower | Upper |
| <1500 | NRBT | 0.036 | 0.520 | 0.282 | 0.959 |
|  | Birth weight | 0.000 | 3.800 | 2.049 | 7.045 |
|  | Birth length | 0.327 | 0.738 | 0.402 | 1.354 |
|  | Head circumference | 0.712 | 0.887 | 0.468 | 1.680 |
|  | No. of ventilated newborns | 0.005 | 2.949 | 1.375 | 6.328 |
|  | Admission Haemoglobin | 0.067 | 0.993 | 0.986 | 1.000 |
|  | No. of RBC transfusion | 0.000 | 0.623 | 0.489 | 0.794 |
| 1500-2500 | NRBT | 0.126 | 1.952 | 0.829 | 4.595 |
|  | Birth weight | 0.014 | 0.363 | 0.162 | 0.814 |
|  | Birth length | 0.641 | 0.829 | 0.376 | 1.825 |
|  | Head circumference | 0.770 | 1.122 | 0.518 | 2.431 |
|  | No. of ventilated newborns | 0.026 | 2.449 | 1.115 | 5.381 |
|  | Admission Haemoglobin | 0.125 | 0.992 | 0.981 | 1.002 |
|  | No. of RBC transfusion | 0.007 | 1.566 | 1.130 | 2.170 |
| >2500 | NRBT | 0.028 | 3.052 | 1.128 | 8.255 |
|  | Birth weight | 0.087 | 0.501 | 0.227 | 1.106 |
|  | Birth length | 0.258 | 1.665 | 0.688 | 4.026 |
|  | Head circumference | 0.530 | 0.770 | 0.340 | 1.741 |
|  | No. of ventilated newborns | 0.000 | 10.046 | 4.140 | 24.379 |
|  | Admission Haemoglobin | 0.184 | 0.992 | 0.979 | 1.004 |
|  | No. of RBC transfusion | 0.672 | 0.888 | 0.511 | 1.542 |
